# The Strehler-Mildvan correlation as a valuable tool for monitoring the long-term health status of a population

**DOI:** 10.3389/fpubh.2025.1627111

**Published:** 2025-10-02

**Authors:** Josef Dolejs

**Affiliations:** University of Hradec Králové, Hradec Králové, Czechia

**Keywords:** public health, mortality rate, aging, Strehler-Mildvan correlation, calendar years

## Abstract

The increase in the logarithm of mortality with age from 40 years onward can be described by a Gompertz linear relationship with two parameters. The long-term relationship between these two parameters can itself be described by another linear relationship known as the Strehler–Mildvan (SM) correlation. Long-term data from three countries were evaluated in the context of the SM correlation. The earliest available periods were 1751–1754 for Sweden, 1816–1819 for France, and 1850–1854 for the Netherlands, while the most recent periods were 2020–2021 for France and the Netherlands, and 2020–2023 for Sweden. The best agreement with the SM model was observed in Sweden, and the weakest in France. While the SM correlation model generally describes long-term trends well, it can be significantly disrupted over shorter calendar periods. If we view the population as a dynamic system, then large short-term shocks—such as World War I—can temporarily break the SM correlation. Over time, however, the system tends to return to an equilibrium state in which the SM model becomes applicable again.

## Introduction

Age-specific mortality intensity is one of the key indicators of public health (1–4). Changes in mortality intensity from age 40 onward are well described by the well-known Gompertz relationship (1, 4–12). The Gompertz model expresses the logarithm of mortality intensity as shown in Equation (1).


(1) $$\ln(\mu (x))=\ln(m_{o})+a.x$$

where μ(x) is mortality intensity, x is age, ln(m_o_) and a are two parameters. The assumptions of a standard regression model using the least squares method may not always be satisfied when modeling the dependence of mortality on age-particularly regarding the independence of residuals. Nevertheless, it is still possible to fit a line to the data in the geometric sense and evaluate its fit using the standard coefficient of determination (R^2^). Coefficients of determination calculated for Gompertz-based linear fits frequently exceed 0.99, even across very different populations with widely varying levels of mortality intensity (6–8, 12–16). Such high R^2^ values are also observed as a secondary outcome in the data analyzed in this study.

Socio-economic development, advances in medicine, war, and other societal factors can influence mortality intensity within a population. One of the effective frameworks for describing the long-term evolution of mortality is the Strehler-Mildvan correlation (SM correlation) (1, 5, 17–29). This concept is based on analyzing temporal changes in the parameters of the Gompertz function. Ideally, it represents a rotation of Gompertz lines around a fixed point in time. In such a scenario, the mortality intensity at the age corresponding to the intersection point of these lines remains constant.

Under normal development—such as improvements in public health—this is geometrically represented by a decrease in mortality at younger ages and a simultaneous increase in the slope of the Gompertz line. This behavior has been observed in human populations as well as in other biological species, and has even been validated under controlled laboratory conditions during artificial manipulations of living environments (25–28). When the intersection point occurs at a relatively young age, temporal development may paradoxically result in increased mortality at older ages.

The original work by Strehler and Mildvan provided a theoretical explanation for this phenomenon using a model based on the concept of “vitality” (28). This study presents a descriptive analysis of the SM correlation in three countries over an extended period. It investigates the extent to which the SM correlation holds in the given datasets and explores whether significant patterns can be detected in terms of long-term population changes.

Previous research has shown that major shifts—such as improved living conditions between the 19 and 20th centuries or the introduction of antibiotics after World War II—did not disrupt the validity of the Gompertz relationship (22–27, 29). Instead, they led to quantitative changes in the parameters of the model. Geometrically, the SM correlation can be represented by examining the relationship between the intercept and the slope (parameter a) of the Gompertz Equation (1). In the SM correlation model, this is formally expressed by Equation (2).


(2) $$\begin{array}{l} y=\alpha .x+\ln(\mu_{o})\;\text{and}\;\mathrm{YA}=\alpha .A+\ln(\mu_{o})\\ =>\ln(\mu_{o}(\alpha ))=-A\;*\alpha +\mathrm{YA}\; \end{array}$$

where x is age, ln(m_o_) and a are Gompertz parameters and (A, YA) are coordinates of the intersection of lines. Each individual Gompertz line can be represented as a point, where the x-coordinate corresponds to the slope *α*, and the y-coordinate represents the second parameter ln(m_0_) of the line from Equation (1). If these lines intersect at a common point at age A, with coordinates (A, YA), then they can be said to rotate around that point. In such cases, the relationship in Equation (2) holds for different Gompertz lines, characterized by varying *α* slopes and ln(m_0_) parameters. The values A and YA then act as constants in the SM correlation model. In the SM correlation graph, the negative slope −A corresponds to the age A, representing the point of intersection.

Over time, this typically manifests as a decrease in the ln(m_0_) accompanied by an increase in the *α* slope (1, 5, 17–29). For example, the discovery of a highly effective drug or treatment method may significantly influence population-level mortality intensity. It is important to note that such changes often follow the SM correlation mechanism. In an extreme case, the SM correlation could even capture the impact of a major medical breakthrough—such as the historical introduction of antibiotics-which fundamentally altered mortality trends, particularly in relation to infectious diseases.

The central hypothesis of this study was that while the SM correlation broadly characterizes long-term mortality trends, it may be temporarily disrupted by significant socio-political or epidemiological events. It is aimed to identify such deviations and assess whether and how populations return to equilibrium patterns over time.

## Methods

Data were downloaded for three countries—Sweden, France, and the Netherlands—from the public Human Mortality Database (30). Mortality intensity was described using five-year periods and one-year age categories. In demographic notation, the data are denoted as 1×5 (age × period). The first available periods were 1751–1754 for Sweden, 1816–1819 for France, and 1850–1854 for the Netherlands, while the most recent period was 2020–2021 for France and the Netherlands, and 2020–2023 for Sweden (the latter corresponds to the COVID years and they are not 5-year). The “Total” dataset, representing the combined data for males and females, was used.

Gompertz parameters were estimated using the least squares method over the age interval 40–95 years. For each calendar period, the parameters from Equation (1) and the coefficient of determination (R^2^) were calculated using the R software (version 4.3.3, ^©^ 2024 The R Foundation for Statistical Computing). Although the residuals are not normally distributed and exhibit a U-shaped pattern (it is characteristic in mortality data), the least squares estimates of the two parameters are used solely for point estimation, without drawing any statistical inference. This approach is discussed in more detail in the study by Dolejs and Maresova (7).

**Figure 1:**
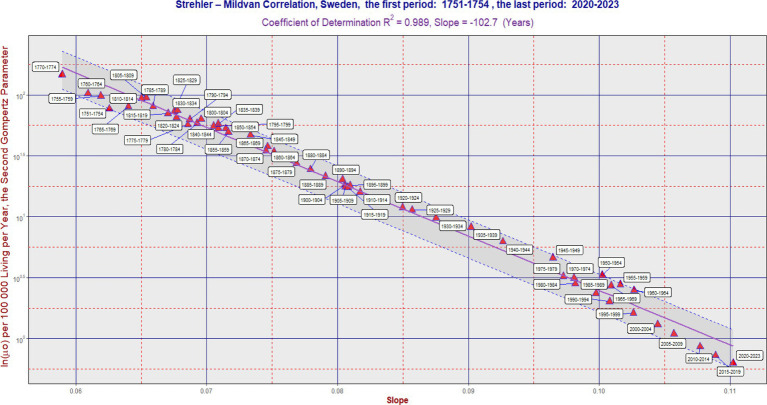
SM correlation in Sweden during the period 1751–2023. The purple line corresponds to Equation (2). The vertical axis is on a logarithmic scale. The band was delimited by the maximum (L2) and minimum residual values (L1). It has the same slope as the violent regression line. The ratio L2/L1 was 2.06 in Sweden. In some cases, the software generated an auxiliary blue line to connect the point with its corresponding calendar period label. Each 5 years calendar period is represented by one point. Table 1 can also be used to better identify the calendar period.

**Figure 2:**
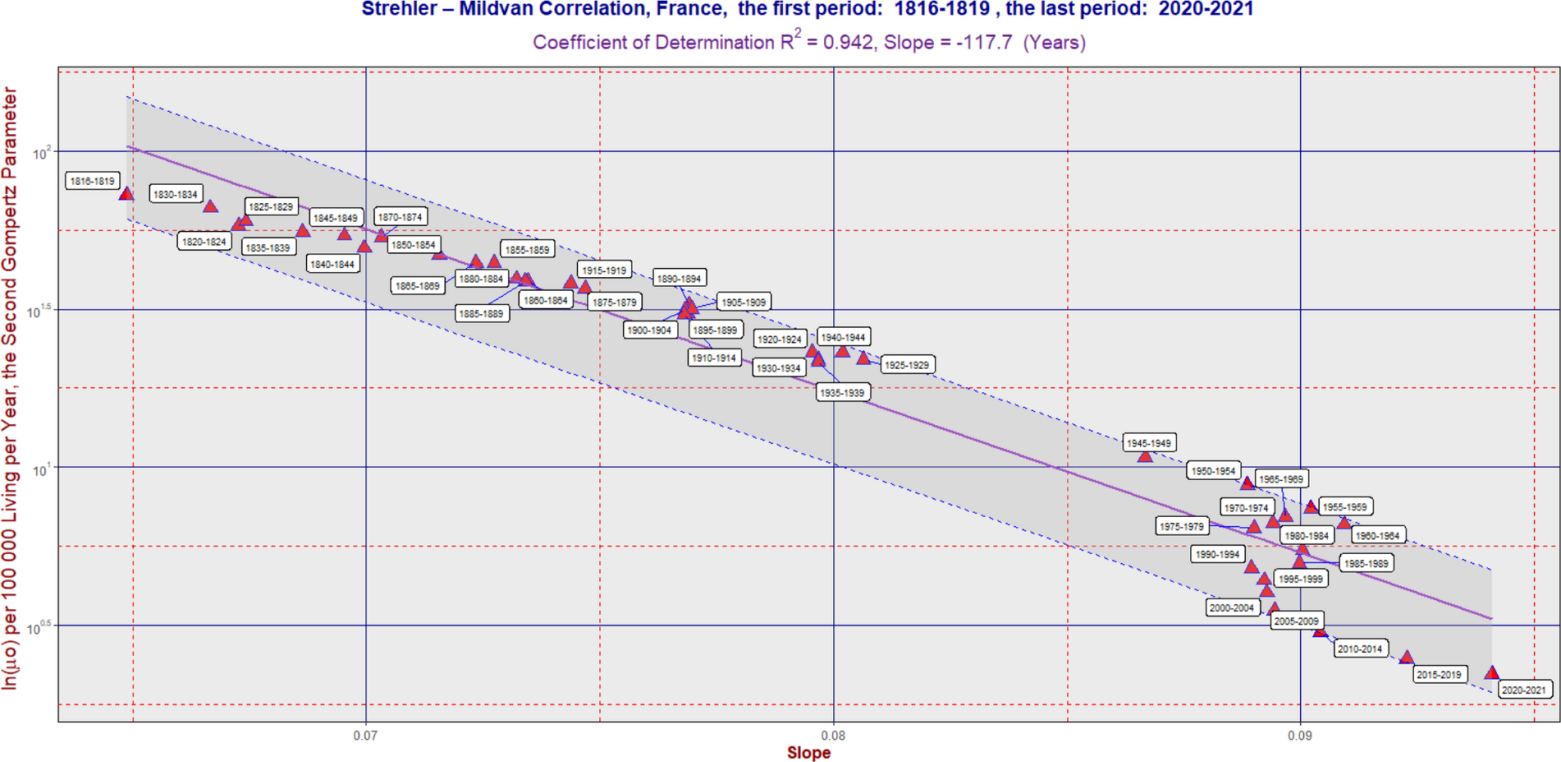
SM correlation in France during the period 1816–2021. The purple line corresponds to Equation (2). The vertical axis is on a logarithmic scale. The band was delimited by the maximum (L2) and minimum residual values (L1). It has the same slope as the violent regression line. The ratio L2/L1 was 2.44 in France. In some cases, the software generated an auxiliary blue line to connect the point with its corresponding calendar period label. Each 5 years calendar period is represented by one point. Table 1 can also be used to better identify the calendar period.

**Figure 3:**
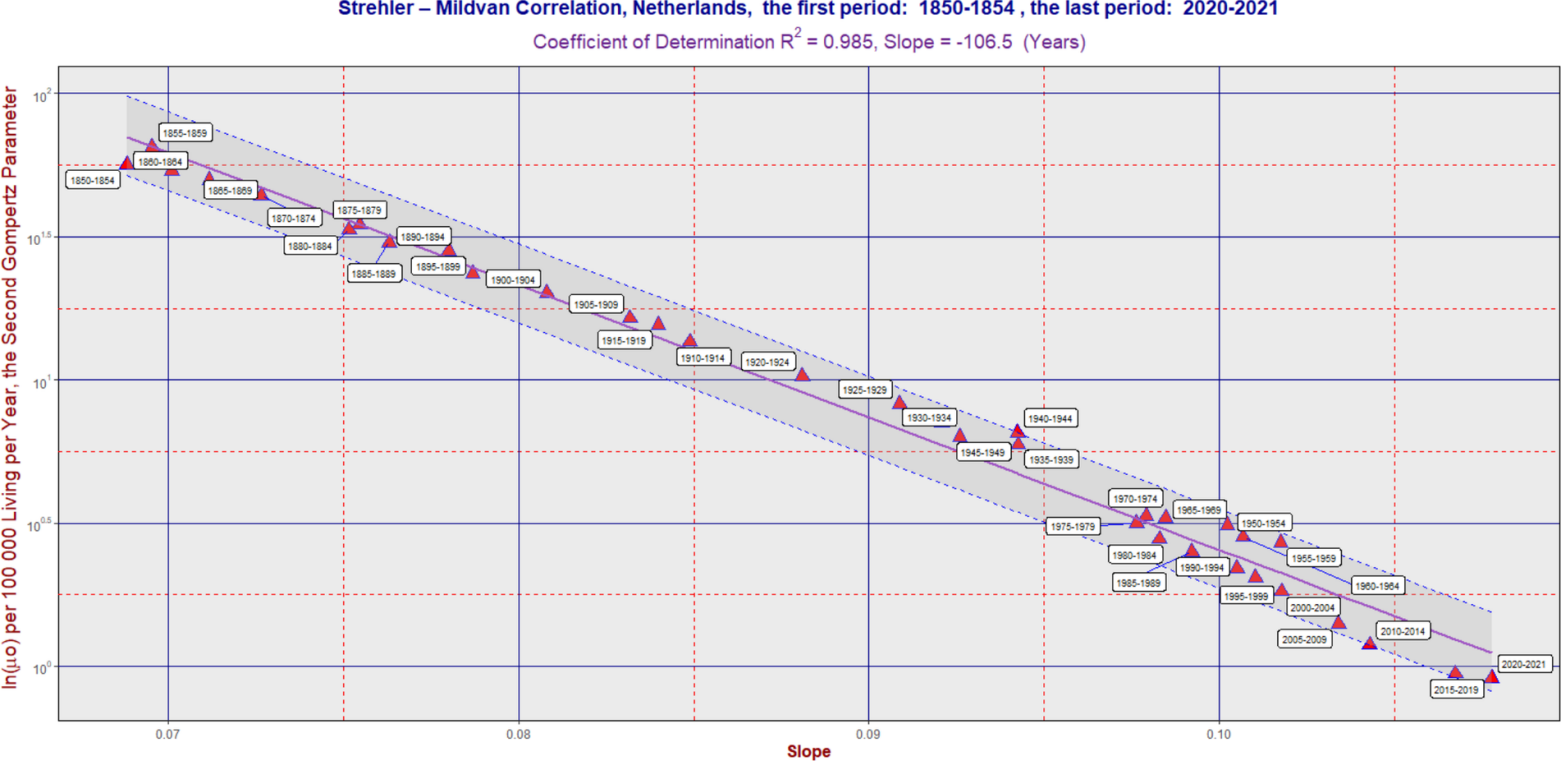
SM correlation in the Netherlands during the period 1850–2021. The purple line corresponds to Equation (2). The vertical axis is on a logarithmic scale. The band was delimited by the maximum (L2) and minimum residual values (L1). It has the same slope as the violent regression line. The ratio L2/L1 was 1.89 in the Netherlands. In some cases, the software generated an auxiliary blue line to connect the point with its corresponding calendar period label. Each 5 years calendar period is represented by one point. Table 1 can also be used to better identify the calendar period.

The numerical results are shown in Table 1. Graphical visualizations in Figures 1–3 were also created using R, specifically the “ggplot2” and “ggrepel” packages. The R code used in this analysis is available upon request from the author.

**Table 1:** Results in three countries.

Sweden						France				
Period	a	1.diff	Sign	ln(mo)	R^2^	a	1.diff	Sign	ln(mo)	R^2^
1751–1754	0.0625	x	x	−7.17	0.9809	x	x	x	x	x
1755–1759	0.0619	−0.0006	N	−6.93	0.9845	x	x	x	x	x
1760–1764	0.0609	−0.0009	N	−6.88	0.9806	x	x	x	x	x
1765–1769	0.064	0.0031		−7.12	0.9838	x	x	x	x	x
1770–1774	0.0589	−0.0051	N	−6.52	0.9832	x	x	x	x	x
1775–1779	0.0685	0.0096		−7.47	0.9859	x	x	x	x	x
1780–1784	0.0692	0.0006		−7.44	0.9842	x	x	x	x	x
1785–1789	0.0659	−0.0033	N	−7.13	0.9857	x	x	x	x	x
1790–1794	0.0687	0.0028		−7.37	0.9871	x	x	x	x	x
1795–1799	0.0705	0.0018		−7.49	0.9876	x	x	x	x	x
1800–1804	0.0695	−0.0009	N	−7.36	0.9919	x	x	x	x	x
1805–1809	0.0653	−0.0042	N	−6.96	0.9946	x	x	x	x	x
1810–1814	0.065	−0.0002	N	−6.96	0.9933	x	x	x	x	x
1815–1819	0.067	0.002		−7.26	0.9895	0.0648	x	x	−7.23	0.9887
1820–1824	0.0676	0.0005		−7.33	0.9906	0.0672	0.0024		−7.45	0.9883
1825–1829	0.0675	−0.0001	N	−7.21	0.9892	0.0674	0.0002		−7.42	0.99
1830–1834	0.0677	0.0001		−7.2	0.9895	0.0666	−0.0007	N	−7.32	0.9877
1835–1839	0.0708	0.0031		−7.45	0.9874	0.0686	0.0019		−7.49	0.9873
1840–1844	0.0708	−0.0001	N	−7.53	0.9891	0.0699	0.0013		−7.61	0.9833
1845–1849	0.0733	0.0025		−7.66	0.9889	0.0695	−0.0003	N	−7.52	0.9858
1850–1854	0.0714	−0.0019	N	−7.54	0.9858	0.0715	0.0019		−7.66	0.9884
1855–1859	0.0716	0.0001		−7.61	0.9888	0.0727	0.0012		−7.72	0.9884
1860–1864	0.0751	0.0035		−7.98	0.9921	0.0734	0.0007		−7.85	0.9899
1865–1869	0.0746	−0.0005	N	−7.88	0.9908	0.0723	−0.0011	N	−7.72	0.9886
1870–1874	0.0745	−0.0001	N	−7.96	0.9883	0.0703	−0.002	N	−7.53	0.9838
1875–1879	0.0768	0.0023		−8.2	0.9863	0.0746	0.0043		−7.91	0.9885
1880–1884	0.0779	0.0011		−8.31	0.9858	0.0732	−0.0014	N	−7.83	0.987
1885–1889	0.079	0.0011		−8.44	0.9831	0.0734	0.0002		−7.86	0.9865
1890–1894	0.0803	0.0013		−8.5	0.9835	0.0769	0.0034		−8.02	0.9874
1895–1899	0.0809	0.0006		−8.62	0.98	0.0768	−0.0001	N	−8.08	0.9872
1900–1904	0.0805	−0.0003	N	−8.6	0.9805	0.0768	0.0001	N	−8.06	0.9881
1905–1909	0.0805	0.0001		−8.64	0.9809	0.0769	0.0001		−8.05	0.9897
1910–1914	0.0817	0.0011		−8.74	0.9837	0.0768	−0.0001	N	−8.09	0.9872
1915–1919	0.0807	−0.001	N	−8.65	0.9809	0.0743	−0.0024	N	−7.87	0.9723
1920–1924	0.0849	0.0042		−9.04	0.9878	0.0795	0.0052		−8.37	0.992
1925–1929	0.0856	0.0006		−9.08	0.9903	0.0806	0.0011		−8.42	0.9913
1930–1934	0.0875	0.0019		−9.23	0.9925	0.0796	−0.001	N	−8.42	0.9932
1935–1939	0.0901	0.0026		−9.4	0.9944	0.0796	0.0001		−8.44	0.9936
1940–1944	0.0926	0.0025		−9.68	0.9961	0.0801	0.0005		−8.37	0.9844
1945–1949	0.0964	0.0038		−9.98	0.9981	0.0866	0.0064		−9.13	0.9951
1950–1954	0.1002	0.0038		−10.32	0.9989	0.0888	0.0022		−9.33	0.997
1955–1959	0.1016	0.0014		−10.49	0.9991	0.0902	0.0014		−9.51	0.9978
1960–1964	0.1026	0.001		−10.6	0.9992	0.0909	0.0006		−9.62	0.9983
1965–1969	0.1009	−0.0016	N	−10.51	0.9986	0.0896	−0.0013	N	−9.57	0.9987
1970–1974	0.098	−0.0029	N	−10.37	0.9987	0.0894	−0.0002	N	−9.61	0.9978
1975–1979	0.0972	−0.0008	N	−10.33	0.9986	0.0889	−0.0004	N	−9.65	0.9966
1980–1984	0.0981	0.0009		−10.47	0.9991	0.09	0.001		−9.8	0.9952
1985–1989	0.0997	0.0015		−10.65	0.999	0.0899	−0.0001	N	−9.9	0.9944
1990–1994	0.1007	0.001		−10.8	0.9986	0.0889	−0.0009	N	−9.94	0.9918
1995–1999	0.1026	0.0019		−11.03	0.9983	0.0892	0.0002		−10.03	0.9905
2000–2004	0.1044	0.0018		−11.24	0.9978	0.0892	0.0001		−10.12	0.9872
2005–2009	0.1057	0.0013		−11.42	0.9972	0.0894	0.0001		−10.24	0.9863
2010–2014	0.1077	0.002		−11.66	0.9972	0.0904	0.001		−10.41	0.9866
2015–2019	0.1088	0.001		−11.83	0.9965	0.0922	0.0018		−10.6	0.9882
2020–2023*	0.1102	0.0014		−11.97	0.9966	0.094	0.0018		−10.71	0.9913

The Netherlands					
Period	a	1.diff	Sign	ln(mo)	R^2^
1850–1854	0.0688	x	x	−7.47	0.9864
1855–1859	0.0695	0.0007		−7.33	0.9863
1860–1864	0.07	0.0005		−7.53	0.9852
1865–1869	0.0711	0.001		−7.6	0.9842
1870–1874	0.0726	0.0015		−7.72	0.9855
1875–1879	0.0754	0.0028		−7.96	0.9873
1880–1884	0.0751	−0.0002	N	−8	0.9873
1885–1889	0.0763	0.0012		−8.1	0.9877
1890–1894	0.0779	0.0015		−8.17	0.9898
1895–1899	0.0786	0.0007		−8.35	0.9916
1900–1904	0.0808	0.0021		−8.51	0.9923
1905–1909	0.0831	0.0023		−8.71	0.9947
1910–1914	0.0848	0.0017		−8.9	0.9956
1915–1919	0.0839	−0.0008	N	−8.76	0.9929
1920–1924	0.088	0.004		−9.17	0.9968
1925–1929	0.0908	0.0028		−9.4	0.9973
1930–1934	0.092	0.0011		−9.55	0.9982
1935–1939	0.0942	0.0022		−9.72	0.9984
1940–1944	0.0942	−0.0001	N	−9.63	0.9946
1945–1949	0.0925	−0.0017	N	−9.66	0.9965
1950–1954	0.1002	0.0077		−10.37	0.999
1955–1959	0.1017	0.0015		−10.51	0.9993
1960–1964	0.1006	−0.0011	N	−10.47	0.9997
1965–1969	0.0984	−0.0021	N	−10.32	0.9998
1970–1974	0.0979	−0.0005	N	−10.3	0.9998
1975–1979	0.0976	−0.0002	N	−10.36	0.9997
1980–1984	0.0982	0.0005		−10.48	0.9996
1985–1989	0.0992	0.001		−10.59	0.9996
1990–1994	0.1005	0.0013		−10.72	0.9998
1995–1999	0.101	0.0005		−10.79	0.9995
2000–2004	0.1017	0.0006		−10.91	0.9985
2005–2009	0.1034	0.0017		−11.17	0.998
2010–2014	0.1042	0.0007		−11.34	0.9974
2015–2019	0.1067	0.0025		−11.57	0.9969
2020–2021*	0.1077	0.001		−11.61	0.9968

*The last period 2020–2021 was in France and Netherlands and the last period 2020–2023 was in Sweden. The symbols a and ln(m_o_) are Gompertz parameters calculated in Equation (1) with the standard coefficient of determination R^2^ (the last column of country). “1.diff” is the difference between the slope a in specific row and the previous slope a. Symbol “N” simply denotes negative value of “1.diff”.

## Results

In the long term, the SM correlation was confirmed in all three countries, as illustrated in Figures 1–3. Numerically, the SM correlation was evaluated using the least squares method. In Sweden, the slope in Equation (2) was calculated to be −102.7 years, with a coefficient of determination (R^2^) of 0.989; in France, −117.7 years, with the lowest R^2^ of 0.942; and in the Netherlands, −106.5 years, with an R^2^ of 0.985. The bands delimited by the maximum (L2) and minimum residual values (L1) are shown in Figures 1–3. These bands have a fixed slope and a width corresponding to the ratio of the maximum to the minimum residual values (note: the y-axis in Figures 1–3 is logarithmic). The L2/L1 ratios, which represent the width of these bands, are as follows: 2.06 in Sweden, 2.44 in France, and 1.89 in the Netherlands.

A poorer fit of the data to the SM model was observed in France, primarily associated with developments in the second half of the 20th century. During this period, the slope did not change significantly, while the second parameter gradually decreased over time (see period: 1950–2004). This is reflected both in the lowest value of the coefficient of determination and the widest confidence band (i.e., the highest L2/L1 ratio). A more detailed numerical evaluation of the longitudinal development is provided in Table 1.

In the “Sign” columns, the symbol “N” denotes instances where the first difference of the slope (“1.diff”) over time was negative (i.e., the slope value was lower than in the preceding period). This indicates that the change in slope was not consistent with the SM correlation model. Notably, Table 1 shows periods where the symbol “N” appears simultaneously for all three countries—specifically, during the periods “1965–1969,” “1970–1974,” and “1975–1979.” A similar result across all three countries is also found in the period “1915–1919,” corresponding to the First World War. In contrast, during the most recent period (the COVID period), no negative trend changes were observed.

Figures 1–3 show several periods where the observed developments deviated from the main trend predicted by the SM model, as well as periods where the changes aligned with it. For example, during the second half of the twentieth century, the trend in France did not follow the SM model. Instead, there was a primarily vertical shift caused by a decrease in the parameter ln(*μ*₀), reflecting a positive development marked by declining mortality across most age categories, without significant changes in the slope itself. In more recent periods, however, the trend in France has returned to alignment with the SM model. In fact, the development in all three countries studied has closely followed the SM correlation model in recent decades.

## Discussion

The data used and the results derived from them cover a period of 273 years in Sweden, 206 years in France, and 172 years in the Netherlands, providing unique insights into the long-term development of mortality intensity. The SM correlation model describes the behavior of Gompertz parameters over extended periods. The model showed the least success in France, primarily due to trends observed in the second half of the 20th century. Interestingly, from 2000 to 2021, the development in France aligned more closely with the SM correlation model.

One possible interpretation of the observed results is that the population may be viewed as a kind of closed system in which the SM correlation dominates in the long term. When the system is exposed to a strong external influence—such as during the period 1915–1919 (World War I) or the three consecutive periods 1965–1969, 1970–1974, and 1975-1979—it eventually returns to an equilibrium state consistent with the SM correlation model. The nature of the external influence during the three consecutive periods mentioned should be investigated in more detail to better understand its impact.

The SM correlation model and its application to data represent an important tool for understanding long-term trends in public health. It also provides a framework for explaining changes in the spectrum of disease causes, particularly the emergence of entirely new disease categories in older age (4, 13, 18).

If we consider a hypothetical scenario in which a highly effective treatment is introduced for a complex group of diseases—such as malignant neoplasms or cardiovascular disorders, similar to the decline in infectious disease mortality in Europe during the 20th century—then, according to the SM correlation model, we would expect an increase in the slope of the Gompertz model and a decrease in the second parameter, ln(m_0_). This would likely be accompanied by a significant shift in the distribution of causes of death.

These findings have significant implications for public health planning (31–34). The ability of the SM model to reflect long-term mortality equilibrium allows it to serve as a benchmark for identifying deviations due to public health crises or interventions (32, 34). As cause-of-death distributions shift toward chronic and neurodegenerative diseases, understanding SM dynamics could inform the allocation of resources, monitoring of aging trajectories, and evaluation of long-term health system performance (34–37). In particular, recognizing early signs of systemic disruption may improve preparedness for future demographic transitions.

Prior work on healthy aging [e.g., (34–36)] has emphasized the importance of tracking not only mortality rates but also shifts in morbidity and functional health (34–36). Our findings suggest that the SM correlation may indirectly capture elements of these transitions by reflecting the evolving mortality dynamics associated with aging populations.

This study offers an innovative approach by applying the SM correlation framework to a unique, long-term, multi-country dataset and interpreting deviations in relation to systemic shocks. Unlike previous studies, it examines not only the validity of the SM model over time but also the dynamic re-equilibration of populations after large-scale events, offering new perspectives on the resilience of demographic systems (31, 32).

## Data Availability

Publicly available datasets were analyzed in this study. This data can be found here: Human Mortality Database (2024), available online at: https://www.mortality.org/.
